# Supplementary figures and images for: Eutrophication trends in the coastal region of the Great Tokyo area based on long-term trends of Secchi depth
Source: PeerJ. 2023 Jul 28;11:e15764. doi: 10.7717/peerj.15764 (PMC10389074; doi:10.7717/peerj.15764)

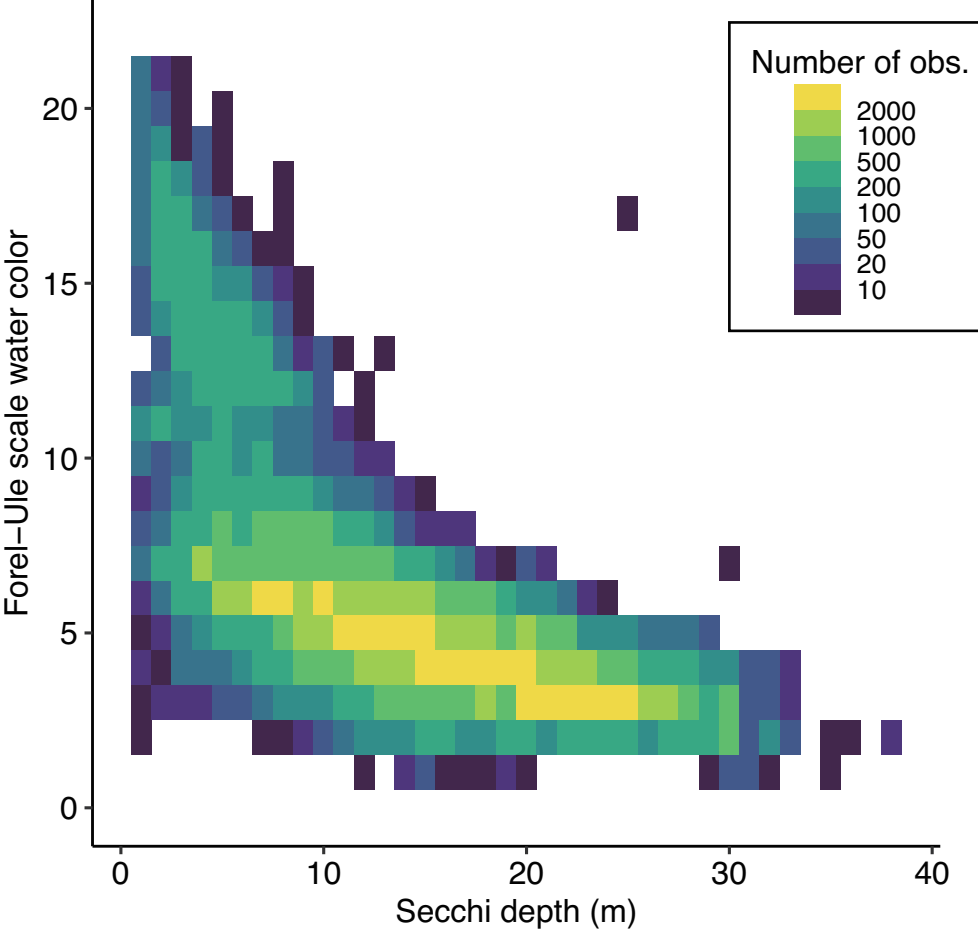

Supplement: Supplemental Information 1 — The color indicated the number of observations. [file peerj-11-15764-s001.pdf]

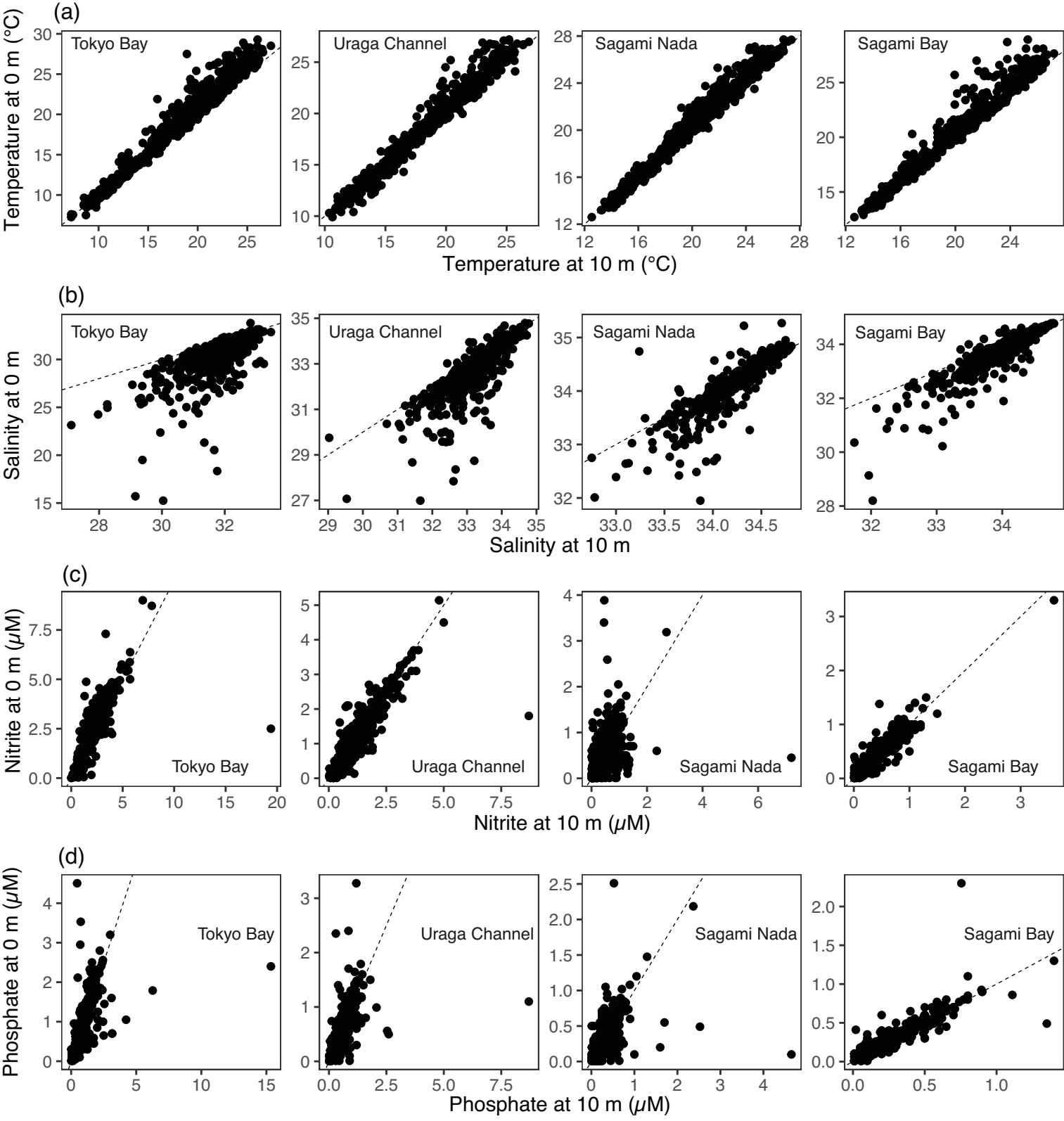

Supplement: Supplemental Information 2 — (a) temperature at the surface and 10 m depth, (b) salinity at the surface and 10 m depth, (c) nitrite concentration at the surface and 10 m depth, and (d) phosphate concentration at the surface and 10 m depth in Tokyo Bay (left), Uraga Channel (second left), Sagami Nada (second right), and Sagami Bay (right). The dotted line is 1:1 line. [file peerj-11-15764-s002.pdf]

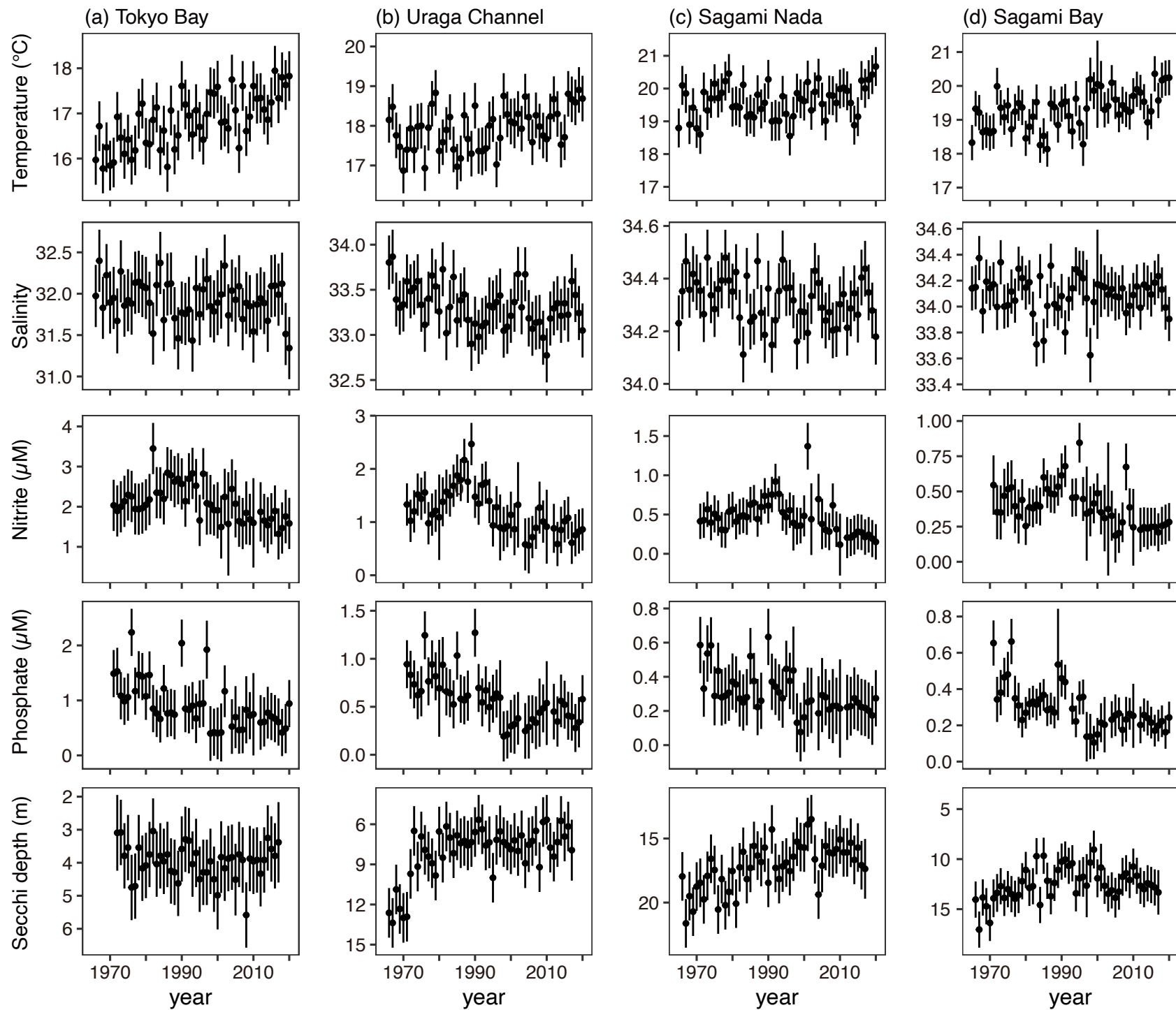

Supplement: Supplemental Information 3 — (a) Tokyo Bay, (b) the Uraga Channel, (c) Sagami Nada, and (d) Sagami Bay. The parameters except Secchi depth was collected at 10 m depth. The closed circle and vertical bar denote the yearly least squared mean and standard errors, respectively in every subarea. The regression line was not shown. The least squared mean values were calculated from a model whose description was a following equation: parameters ~ lm(f.year + f.month). Here, f.year and f.month denote year transformed as a categorical value, and month transformed as a categorical value, respectively. The “parameter” were median values of temperature, salinity, nitrite concentration, phosphate concentration, and Secchi depth in every observation of every subarea. [file peerj-11-15764-s003.pdf]

Coefficient

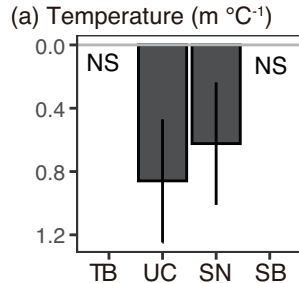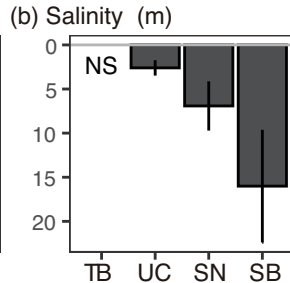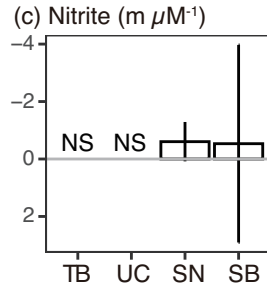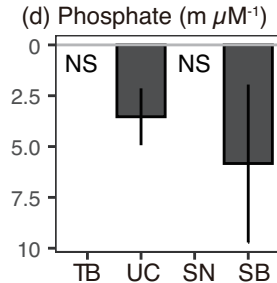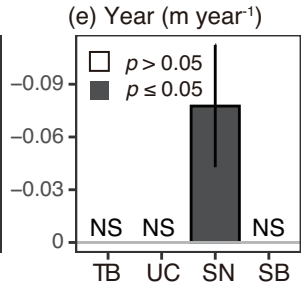

Subareas

Supplement: Supplemental Information 4 — (a) temperature, (b) salinity, (c) nitrite, (d) phosphate, and (e) year in Tokyo Bay (TB), the Uraga Channel (UC), Sagami Nada (SN), and Sagami Bay (SB) in a linear model with a Prais-Winsten estimator. The bar height is the coefficient value, and the bar denotes the 95% confidence interval. NS shows that the parameter was not selected in the least-AIC model. [file peerj-11-15764-s004.pdf]
